# Enlarging the Toolbox Against Antimicrobial Resistance: Aptamers and CRISPR-Cas

**DOI:** 10.3389/fmicb.2021.606360

**Published:** 2021-02-19

**Authors:** Higor Sette Pereira, Thaysa Leite Tagliaferri, Tiago Antônio de Oliveira Mendes

**Affiliations:** Laboratory of Synthetic Biology and Modelling of Biological Systems, Department of Biochemistry and Molecular Biology, Universidade Federal de Viçosa, Viçosa, Brazil

**Keywords:** antimicrobial resistance, molecular diagnostic, alternative treatments, aptamer, CRISPR-Cas

## Abstract

In the post-genomic era, molecular treatments and diagnostics have been envisioned as powerful techniques to tackle the antimicrobial resistance (AMR) crisis. Among the molecular approaches, aptamers and CRISPR-Cas have gained support due to their practicality, sensibility, and flexibility to interact with a variety of extra- and intracellular targets. Those characteristics enabled the development of quick and onsite diagnostic tools as well as alternative treatments for pan-resistant bacterial infections. Even with such potential, more studies are necessary to pave the way for their successful use against AMR. In this review, we highlight those two robust techniques and encourage researchers to refine them toward AMR. Also, we describe how aptamers and CRISPR-Cas can work together with the current diagnostic and treatment toolbox.

## Antibiotic Resistance Crisis

Despite antimicrobials’ impact on modern medicine since their introduction in the first part of the 19th century (Powers, 2004; CDC, 2019), resistant bacteria quickly emerged throughout the decades. Drug resistance to all available antibiotics has been detected in clinical bacteria, threatening all advances achieved within the antibiotic era and urging for alternative treatments (CDC, 2019).

Bacteria have developed resistance mechanisms to avoid, disrupt, eject, or resist the currently used antimicrobials (Box 1). They can be intrinsically resistant to antibiotics by using structural or functional inherent bacterial features or acquire resistance via genetic mutations or by horizontal transference of genetic elements (Blair et al., 2015; Munita and Arias, 2016).

The spread of bacterial resistance mechanisms has been much faster than the development of new treatments. New investments on antimicrobial research have been discouraged due to their elevated production costs and long-term development process (Adams and Brantner, 2010). On top of that, the misuse and over-prescription of antibiotics, which stem from uncertainties in diagnosis, contribute to the antimicrobial resistance (AMR) crisis escalation (Llor and Bjerrum, 2014; Malik and Bhattacharyya, 2019). The lack of rapid diagnostic tools directly affects initial treatment decisions, which might lead to empirical treatment guided only by clinical presentation (Fischer et al., 2004; Leekha et al., 2011).

Phenotypic-based diagnostics are currently considered as gold standards in AMR assessment. The “catch-all” resistance characteristic of phenotypic tests enables the evaluation of microbial susceptibility in a relatively unbiased way (Mitsakakis et al., 2018). Although efforts have been made to provide quick phenotypic tests (∼7 h) (Pancholi et al., 2018) to better guide antibiotic treatment, the most used techniques still require microorganism culture, with a turnaround time of around 18 h. This delays the availability of the AMR profiles, which might be accessible up to 72 h after sample collection (Leekha et al., 2011). Besides time-to-result limitation, phenotypic tests generally require laboratory structure (Mitsakakis et al., 2018). Therefore, quicker and accessible diagnostic tools are imperative to guide the first medical decisions regarding antimicrobial therapy prescription worldwide.

## Molecular Approaches

The search for more precise molecular diagnostic tools with quicker turnaround times has been encouraged to better guide clinical practice and public health policies. The recent global SARS-CoV-2 (severe acute respiratory syndrome coronavirus 2) outbreak has shown that in a matter of weeks, diagnostic centers have been overloaded with patients’ samples and quick result release is required for viral spread control. So far, until 21 December 2020, SARS-CoV-2 virus infected 75,704,857 individuals, with 1,690,061 deaths worldwide^1^. We dare to draw here a parallel between SARS-CoV-2 and the AMR crisis. Currently, AMR infections cause around 700,000 deaths per year (O’Neil, 2016). In both cases, an early diagnosis would give trustworthy information for discrimination and contention of the causative agent. With alarming death numbers, the exploration of alternative treatments and diagnostics comes into the spotlight as an attempt to revert the current scenario caused by AMR.

Different molecular tools have been employed as a diagnostic to identify infectious disease agents and their resistance profile. RT-qPCR (quantitative reverse transcription PCR) and NGS (Next-Generation Sequencing) have been currently playing a key role in the diagnostics of the SARS-CoV-2, different from what happened in the 2002 SARS outbreak (Sheridan, 2020). qPCR is also an outstanding tool for the molecular detection of antimicrobial resistance genes (ARGs) (Waseem et al., 2019) directly from patient samples such as urine, blood, and cerebrospinal fluid (Singh et al., 2017). In addition to qPCR, metagenomic, LAMP (Loop-mediated isothermal amplification), and whole genome sequencing approaches not only characterize pathogens at the species level but also detect ARGs (Zankari et al., 2012; Dekker, 2018; Ota et al., 2019).

The molecular diagnostic tools described above offer an abundant panel to recognize DNA and RNA of infectious microorganisms. Complementarily, proteomics- and metabolomics-based techniques have been gaining momentum into the clinical molecular diagnostic field, for instance, MALDI-TOF MS (Matrix-Assisted Laser Desorption/Ionization Time-of-Flight Mass Spectrometry) (Patrinos et al., 2017). However, these tools require expensive equipment and laboratory structure, hampering their wide implementation as *in loco* diagnostic tools.

**Box 1. Antimicrobial resistance mechanism.**

**FIGURE 1:**
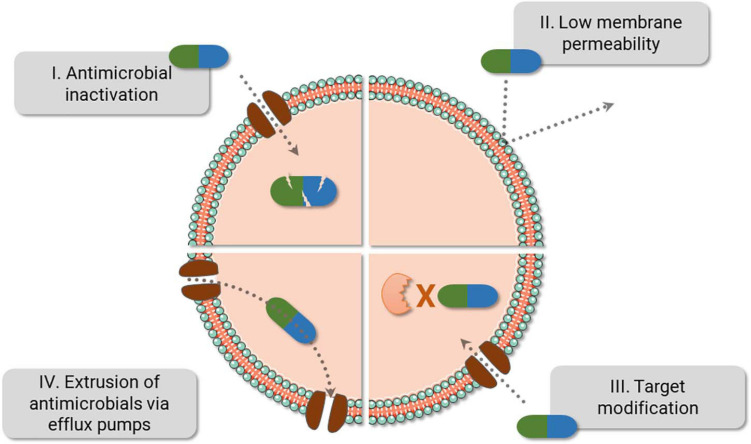
Bacteria fight back antimicrobials. The main bacterial resistance mechanisms are displayed above. Of note, resistance against one class of antibiotics can be achieved using different strategies. For instance, resistance against beta-lactams can be caused by PBP2a (III), beta-lactamase (I), or by a combination of different mechanisms, i.e., reduction of porins (II) and the use of beta-lactamase (I). Also, efflux pumps can expel several antibiotic classes (IV) and are frequently present in multidrug resistance bacteria.

Resistance mechanisms date back to thousands of years and have been probably used to endure the presence of toxic compounds present in nature—including antimicrobials derived from different microorganisms, while they also provide alternative cellular functions (Allen et al., 2010; D’costa et al., 2011). Bacteria can evade antimicrobials via reduction of drug intracellular concentration either by low membrane permeability or through antibiotic efflux; target modification by genetic mutation or post-translational modification; and inactivation of the antibiotic by hydrolysis or its modification (Blair et al., 2015). With the introduction and constant presence of antimicrobials in medical care, agriculture and animal health, the spread of resistant microorganisms and the evolution of their defense strategies have been accelerated (Figure 1; CDC, 2019). From all resistance mechanisms, genes responsible for antibiotic inactivation and target alteration (Figure 1 I and III, respectively) are commonly spread by plasmids and phage transduction (Munita and Arias, 2016; Calero-Caceres et al., 2019). Antibiotic inactivation is a usual strategy adopted for instance against beta-lactams and aminoglycosides. Beta-lactams can be hydrolyzed by enzymes encoded by the *bla* genes (beta-lactamase genes), such as *bla*_*TEM*_, *bla*_*KPC*_, and *bla*_*OXA*_. Aminoglycosides, by its turn, are chemically inactivated by mainly three biochemical reaction, named adenylation, acetylation, and phosphorylation catalyzed by the enzymes nucleotidyltransferases (ANT), acetyltransferases (AAC), and phosphotransferases (APH), respectively (Doi et al., 2016; Munita and Arias, 2016; Bush and Bradford, 2019). An advantage of the hydrolysis over the chemical alteration strategy is the requirement of water instead of chemical compounds as a co-substrate, which ease enzyme activity outside the cell. Target alteration can be achieved by four main strategies, affecting several antimicrobials (not limited to the examples), as follows: (i) Target protection. One of the best-studied examples involves the determinants Tet(M) and Tet(O), which confers resistance to tetracycline. They interact with the ribosome and dislodge the drug from its binding site. (ii) Mutation of the antimicrobial target site. The development of mutations in the chromosomal genes *gyrA-gyrB* and *parC-parE* codifying for DNA gyrase and topoisomerase IV, respectively, promotes resistance against fluoroquinolones. (iii) Enzymatic alteration. Macrolide resistance is acquired by *erm* genes (erythromycin ribosomal methylation), which codify enzymes responsible for 50S ribosomal subunit methylation. This alteration weakens the binding of the erythromycin to the ribosome. (iv) Replacement/bypass of the target. Beta-lactam resistance is frequently acquired by Gram-positive microorganisms via *mec*A gene. The gene encodes an exogen penicillin-binding protein (PBP2a) that has low affinity for the beta-lactams, opposite to what happens to endogen PBPs (Munita and Arias, 2016).

Aptamers and CRISPR-Cas (clustered repetitive interspaced short palindromic repeats, CRISPR-associated enzymes) systems have been slowly gaining support in clinical diagnosis and treatment of infectious diseases. Both can be employed as an onsite diagnostic tool with a quick turnaround time, which makes them more interesting than other methods targeting proteins or nucleic acids. Therefore, reviewing these two robust techniques attempts to encourage molecular biologist researchers to develop and refine clinical molecular tools against ARGs. Those methods do not necessarily intend to substitute the already implemented diagnostic approaches, but to stimulate their combination to circumvent antibiotic misuse. Also, their application in therapy will be reviewed to help paving the way for their use as treatments against multidrug-resistant agents.

## Application of Aptamers Into Detection and Neutralization of AMR Factors

**FIGURE 2:**
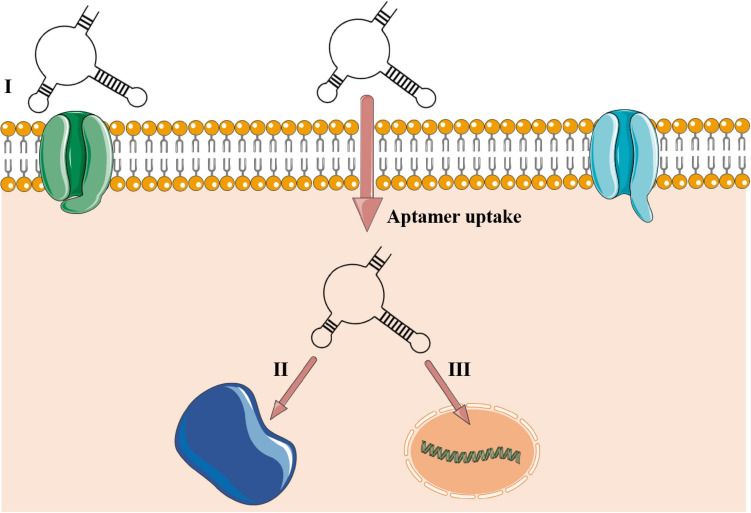
Aptamer–target interactions in extra- and intracellular environments. (I) Aptamer recognizing cell membrane receptor or surface protein. The aptamer is uptaken into the cellular environment and can be located in some subcellular compartments such as lysosome, mitochondria, Golgi, and endoplasmic reticulum or guided to the nucleus in eukaryotic cells (Yoon and Rossi, 2018) or be stored into the cytoplasm of prokaryotic cells. (II) Cytosolic intracellular aptamers recognizing circulating proteins. (III) Nuclear intramers interacting directly with genetic elements. Aptamers’ uptake is mediated to the intracellular environment by a clathrin-dependent mechanism. Clathrin is a group of proteins present in membrane cavities that assist in membrane vesicle formation during the molecule internalization by endocytosis.

A substantial boost in aptamer application in research and clinical institutes has been noted worldwide (McKeague et al., 2015). Also called chemical antibodies (Toh et al., 2015), most aptamers interact with their targets in a constant equilibrium with binding affinities up to 1 pM (Ha et al., 2017). They offer a cheap large-scale production with chemical modifications, low or no immunogenicity, small size (close to 3 nm), flexibility in tridimensional structure, and great stability in different conditions of pH, temperature, and organic solvents (Yoon and Rossi, 2018). Also, aptamers interact with high sensitivity toward their targets, being able to discriminate a single amino acid mutation (Chen et al., 2015). Due to their small size, aptamers reach cavities that are often not accessible to monoclonal antibodies and, therefore, penetrate cell tissues more easily (Xiang et al., 2015). In this process, intracellular aptamers could be internalized by either a clathrin-dependent or -independent mechanism and co-localized in subcellular compartments, directly associated to its target, as reviewed in Yoon and Rossi (2018; Figure 2).

Single-stranded oligonucleotides can assume several secondary conformations such as hairpin, loop, pseudoknot, and G-quadruplex, which assure a unique folding for each sequence and allow interaction with specific sites (Yunn et al., 2015). DNA and RNA aptamers have similar binding characteristics, although DNA nucleotides have lower operating cost and offer greater stability than RNA, which in turn have greater versatility in their three-dimensional structures that directly affect target affinity.

Aptamer selection commonly occurs via a randomized process of systematic evolution of ligands by exponential enrichment (SELEX), firstly reported in 1990 (Ellington and Szostak, 1990; Tuerk and Gold, 1990). SELEX is based on iterative rounds of incubation of the oligonucleotide pool with the target, frequently divided into four stages: incubation, partition, recovery, and amplification. Also, aptamer–target affinity slowly increases alongside the rounds until the most specific aptamer is selected. SELEX allows greater versatility of binding conditions, which favors the adaptation of selected oligonucleotides to different cellular and non-cellular environments (McKeague et al., 2015).

Aptamer selection against many biological and chemical targets has been described (Qiao et al., 2018; Dalirirad and Steckl, 2020), along with its use in drug development (Esposito et al., 2018), bioimaging (Kim et al., 2019), food inspection (Duan et al., 2016), genetic modulation (Mol et al., 2019), and as a delivery vehicle (Zhuang et al., 2020). In antiviral therapy, G-3 aptamer dually inhibits HIV-1 cell replication both by blocking virus entrance via CCR5 receptor and by delivering a siRNA that decreases HIV-1 cytoplasmatic traffic (Zhou et al., 2015). Also, aptamers blocked quorum sensing and inhibited biofilm formation in *Pseudomonas aeruginosa* infections (Zhao et al., 2019). Even with many progresses, there is currently only one aptamer approved for clinical use, named pegaptanib. It is the main component of Macugen^®^, released by the FDA in 2004 to treat age-related macular degeneration (Gragoudas et al., 2004). Recently, there have been 10 therapeutic aptamers at different stages of clinical trials (Kaur et al., 2018), most of them employed to inhibit protein–protein interaction or act as antagonist (Yunn et al., 2015). To date, there is no aptamer currently approved by FDA for diagnostic purposes, even though they fit the quality standards of the diagnostic industry: affordable, sensitive, specific, user-friendly, and robust.

Different diagnostic devices based on aptamers have been proposed, including Aptamer-Linked Immobilized Sorbent Assay (ALISA), dot-blot, lateral-flow strips conjugated to nanomaterials, and the promising aptamer-based sensors (Stoltenburg et al., 2016; Shin et al., 2018; Su et al., 2018; Xiong et al., 2020). Aptasensors can be conjugated to a wide diversity of reporter molecules without modification of their activity (McKeague et al., 2015). Signal transducers commonly conjugated to aptasensors include but are not limited to colorimetric, electrochemical, and fluorescent approaches (Bai et al., 2017; Bayrac et al., 2017; Cai et al., 2019). Linking aptamer-based biosensors with nanomaterials can increase specificity and sensitivity of target binding up to 10-fold and offers a platform for rapid point-of-care diagnostic (<1 h) (Dalirirad and Steckl, 2020). Aptasensors have well-established protocols of chemical conjugation of aptamers with color or signal-transductor molecules, such as gold, silver, platinum, iron oxide nanoparticles, or carbon nanotubes and graphene oxide (Duan et al., 2016; Dehghani et al., 2018; Gao et al., 2018; Hua et al., 2018; Das et al., 2019a; Fan et al., 2020). The application of aptasensors for disease diagnosis has been tested in different samples, e.g., plasma and spiked nasal swab (Qiao et al., 2018), cultured bacteria (Maldonado et al., 2020), and urine and serum samples (Su et al., 2018). A lateral-flow paper strip conjugated with a gold nanoparticle aptamer-based sensor was developed to onsite detection of dopamine in urine samples (Dalirirad and Steckl, 2020).

When repurposed to bacteria, aptamers could recognize them by binding to antigens or cell surface receptors, or interacting with the whole cell through unknown targets (Tang et al., 2016; Song M. Y. et al., 2017; Shin et al., 2019). Although there are uncertainties concerning the mechanisms of aptamer uptake in bacteria, a report indicates that aptamers could traffic inside bacterial cells (Afrasiabi et al., 2020), similarly to what is shown in Figure 2. From 2016 to 2020, several papers have reported the use of aptamers applied to diagnostics of bacterial infections (Table 1). Nearly all studies employed biosensors based on DNA aptamers, which indicates that for diagnostic purposes, the DNA stability overcomes the advantage of tridimensional possibilities of RNA. It is worth mentioning that only three of these studies targeted resistant bacteria or products of ARGs. First, Fan et al. (2020) made a graphene-oxide aptasensor based on peroxidase-like activity for the detection of purified PBP2a protein, encoded by the *mec*A gene. Also, Maldonado et al. (2020) employed a fast and label-free photonic pegylated aptasensor that recognizes both pure PBP2a protein and methicillin-resistant *Staphylococcus aureus* (MRSA)-infected cells in culture. Finally, Qiao et al. (2018) detected PBP2a in *S. aureus* cells collected in clinical plasma and spiked nasal swab samples infected with MRSA strains using a single bacterial lysis step. Predominantly, the diagnostic approach using aptamer has been focusing on the detection of the whole cell instead of its biological specific components, such as proteins or toxins. Therefore, due to the well-established protein-SELEX approach, there is still room for finding highly specific aptamers that bind to proteins associated with ARGs and enrich the diagnostic toolbox.

**TABLE 1:** Report of literature aptamers applied in the diagnostic of bacterial infections from 2016 to 2020.

**Target**	**Oligo**	**Binding affinity or LOD**	**References**
*Acinetobacter baumannii* isolates	DNA	7.547 ± 1.353 pM	Rasoulinejad and Gargari, 2016
*Bacillus subtilis, Citrobacter freundii, Escherichia coli, Enterobacter aerogenes, Klebsiella pneumoniae, and Staphylococcus* epidermidis cells	DNA	9.22–38.5 nM	Song M. Y. et al., 2017
*Campylobacter jejuni* cells	DNA	100 CFU ml−1	Dehghani et al., 2018
*Escherichia coli* and *Staphylococcus aureus* pathogenic cells	DNA	100 CFU ml−1	Xu et al., 2018
*Escherichia coli* ATCC cells	DNA	11.97 ± 2.94 nM	Marton et al., 2016
*Escherichia coli* cells	DNA	3 CFU ml−1	Jin et al., 2017
*Escherichia coli* cells	DNA	0.66 CFU ml−1	Hua et al., 2018
*Escherichia coli* O157 cells	DNA	107.6 ± 67.8 pM	Amraee et al., 2017
*Escherichia coli* O157:H7 cells	DNA	1.46 × 103 CFU ml−1	Yu et al., 2018
*Escherichia coli* whole cells	RNA	2 × 104 CFU ml−1	Dua et al., 2016
*Escherichia coli* whole-cells	DNA	102 CFU ml−1	Wu et al., 2017
Glycolipid antigen of *Mycobacterium tuberculosis*	DNA	668 ± 159 nM	Tang et al., 2016
Gram-negative outer membrane vesicles	DNA	25 ng/ml	Shin et al., 2019
HspX protein in tuberculous meningitis	DNA	10 pg	Das et al., 2019b
Listeria monocytogenes cells	DNA	2.5 CFU ml−1	Suh et al., 2018
Methicillin-resistant *Staphylococcus aureus* strains	DNA	1.38 × 103 CFU ml−1	Qiao et al., 2018
MPT64 antigen of *Mycobacterium tuberculosis*	DNA	0.2 fg ml−1	Bai et al., 2017
MPT64 antigen of *Mycobacterium tuberculosis*	DNA	100 CFU ml−1	Li N. et al., 2018
*Mycobacterium tuberculosis* cells	DNA	100 CFU ml−1	Zhang et al., 2017
*Mycobacterium tuberculosis* H37Ra cells	DNA	5.09 ± 1.43 nM	Mozioglu et al., 2016
Mycolactone in Buruli ulcer	RNA	1.59–73.0 μM	Sakyi et al., 2016
Mycoplasma-infected cells	DNA	Not informed	Liu et al., 2019
*Neisseria meningitidis* serogroup B	DNA	28.3–39.1 pM	Mirzakhani et al., 2017
PBP2a detection	DNA	20 nM	Fan et al., 2020
PBP2a in nosocomial infections	DNA	29 CFU ml^–1^	Maldonado et al., 2020
Protein A of *Staphylococcus aureus*	DNA	11.3 nM	Stoltenburg et al., 2016
Protein A of *Staphylococcus aureus*	DNA	10 CFU ml^–1^	Reich et al., 2017
*Pseudomonas aeruginosa* cells	DNA	100 CFU ml^–1^	Gao et al., 2018
*Pseudomonas aeruginosa* cells	DNA	60 CFU ml^–1^	Das et al., 2019a
*Salmonella enterica* serovar *typhimurium* in milk samples	DNA	3.37 × 10^2^ CFU ml^–1^	Zhang et al., 2018
*Salmonella enteritidis* cells	DNA	0.309 μM	Bayrac and Oktem, 2017
*Salmonella enteritis* cells	DNA	25 CFU ml^–1^	Chinnappan et al., 2017
*Salmonella typhimurium* cells	DNA	10 CFU ml^–1^	Duan et al., 2016
*Salmonella typhimurium* cells	DNA	123 ± 23 nM	Lavu et al., 2016
*Salmonella Typhimurium* cells	DNA	1 CFU ml^–1^	Ren et al., 2019
*Salmonella Typhimurium* cells	DNA	80 CFU ml^–1^	Wang et al., 2020
*Salmonella Typhimurium* cells	DNA	10 CFU ml^–1^	Appaturi et al., 2020
*Shigella sonnei* cells	DNA	15.89 ± 1.77 nM	Song M. S. et al., 2017
*Staphylococcus aureus* and *Escherichia coli* cells	DNA	10–2,000 CFU ml^–1^	Shen et al., 2016
*Staphylococcus aureus* cells	DNA	16 CFU ml^–1^	Kurt et al., 2016
*Staphylococcus aureus* cells	DNA	10^3^ CFU ml^–1^	Bayrac et al., 2017
*Staphylococcus aureus* cells	DNA	39 CFU ml^–1^	Cai et al., 2019
*Streptococcus pyogenes* cells	DNA	7 nM	Hamula et al., 2016
*Streptococcus pyogenes* serotype M3 cell	DNA	7.47 ± 1.72 pM	Alfavian et al., 2017
*Vibrio parahaemolyticus* cells	DNA	2.04e^–9^ ± 0.12 M	Ahn et al., 2018
*Vibrio parahaemolyticus* cells	DNA	10 CFU ml^–1^	Sun et al., 2019

**Papers recovered on the PUBMED NCBI website. Binding affinity and Limit of Detection (LOD) were represented by K_*d*_ and CFU counting, respectively.**

## CRISPR-Cas as a Tool Against Antimicrobial-Resistant Pathogens

CRISPR-Cas is a ribonucleoprotein (RNP) prokaryotic complex present in 50% of the bacteria and in most archaea that behaves as a prokaryotic adaptive immune system (Makarova et al., 2015). The system confers protection against mobile genetic elements, i.e., bacteriophages, plasmids, and transposons in three coordinated phases: adaptation, CRISPR RNA (crRNA) biogenesis, and interference (Hille et al., 2018). Nearly all CRISPR-Cas system counts on an ingenious mechanism to prevent self-targeting. This includes the recognition of a short sequence called protospacer adjacent motif (PAM) during adaptation and interference stages, present only in foreign nucleic acids (Mojica et al., 2009; Marraffini and Sontheimer, 2010).

Based mainly on the signature *Cas* genes, the new classification of CRISPR-Cas systems includes two different classes, six types, and 33 subtypes (Makarova et al., 2020). The most widespread class 1 CRISPR-Cas comprises types I, III, and IV. It is characterized by effector complexes with multiple Cas proteins responsible for a coordinated action from pre-crRNA processing to target cleavage. By its turn, class 2 consists of types II, V, and VI, which contains a single-protein effector module able to recognize and cleave the targeting nucleic acid (Table 2; Makarova et al., 2020).

**TABLE 2:** General features of CRISPR-Cas systems based on the most well-characterized subtypes.

**Type**	**Multisubunity RNP complex**	**Single protein**	**Signature enzyme**	**Seed sequence**	**Most common substrates**		**Cleavage**
					**RNA**	**DNA**	
I	X		Cas3	1–5 nt and 7–8 nt		X	Single-stranded DNA cleavage
II		X	Cas9	10–12 nt		X	Blunt double-stranded DNA break
III	X		Cas10	Not defined*	X	X	Specific and non-specific ssRNA cleavage. Double-stranded DNA break
IV	Defective CRISPR-Cas loci typically lacking the effector nuclease and the adaptation module						
V		X	Cas12	∼18 nt		X	Double-stranded DNA break with staggered overhangs, non-specific ssDNA break
VI		X	Cas13	Not defined^#^	X		Specific and non-specific ssRNA cleavage

Seed sequence: PAM-proximal sequence in which full complementarity is required for CRISPR-Cas interference. As (i) some CRISPR-Cas subtypes have promiscuous PAM sequences, (ii) the site sequence varies among the subtypes; the PAM sequences have not been displayed in this table. Gleditzsch et al. (2019) review the PAM recognition strategies for all CRISPR-Cas types and engineering approaches to change the PAM recognition sequence. *Inconsistent conclusions regarding the seed region of CRISPR type III: absence of seed or its presence in the 5′ end (Cao et al., 2016) or 3’ end (Peng et al., 2015). ^#^Central seed region proposed for Cas13a (Abudayyeh et al., 2016; Liu et al., 2017). Table based on Semenova et al. (2011); Chen et al. (2018), Hille et al. (2018), and Makarova et al. (2020).

Upon unraveling the CRISPR-Cas potential of gene editing in an easier, cheaper, and flexible way compared to previously established tools [for instance, TALENs (transcription activator-like effector nucleases) and Zinc-finger nucleases], the systems have been quickly repurposed to the biomedical and biotechnology fields (Chen and Gao, 2014; Liu et al., 2018; Zhang et al., 2019; Li et al., 2020). Class 2 CRISPR-Cas has been an attractive option for gene editing as a result of the effector module’s simpler architecture when compared to class 1 (Makarova et al., 2020). Target specificity and cutting activity of the nucleases can be virtually programmed to any gene of interest by means of the short-length crRNA sequence. The engineering of a single-guide RNA (sgRNA) by fusing Cas9 crRNA and tracrRNA was a benchmark for gene editing (Jinek et al., 2012), but off-target effects still hold back CRISPR-Cas full potential. Several studies have tried to overcome this drawback by employing a plethora of modifications to increase the system specificity for gene editing (Kleinstiver et al., 2016; Slaymaker et al., 2016; Chen et al., 2017; Casini et al., 2018; Lee et al., 2018). Similarly, collateral effects of some Cas enzymes, i.e., the ability to indiscriminately (*trans-*) cleave ssDNA/ssRNA unleashed by site-specific DNA/RNA (*cis*-) bound by the crRNA, may also be a limitation for gene editing. However, here, this feature has been exploited for diagnostic purposes.

**FIGURE 3:**
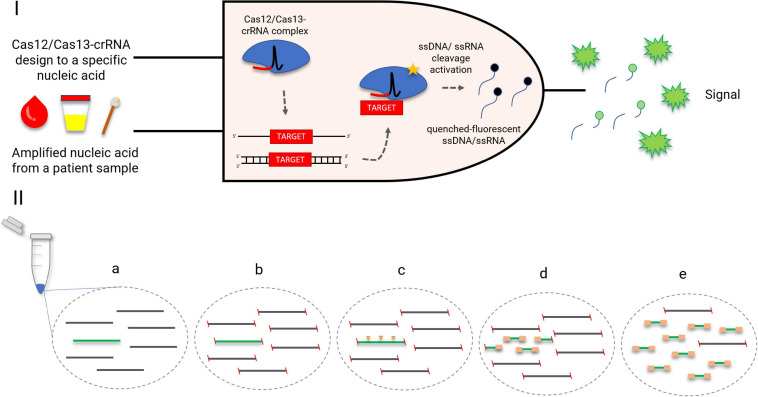
CRISPR-Cas-based diagnostics. (I) A logic gate type “AND” of CRISPR-Cas12/13-based diagnostics. Amplified nucleic acid from patient samples can be used as input. Attomolar concentrations of RNA extracted from serum or urine samples could be detected. Without purification, Cas13 could detect RNA in samples containing up to 2% serum. The diagnostic could be performed also in cfDNA liquid biopsy samples and DNA extracted from anal swabs (Gootenberg et al., 2017; Chen et al., 2018; Gootenberg et al., 2018). A specific crRNA design to match a desired target, for instance, an antimicrobial resistance gene, is coupled with the Cas12/Cas13 as a second necessary input for the diagnostic. Once the target-positive sample is identified by the Cas12/Cas13-crRNA complex, the fluorescent output is generated. The fluorescent signal is a result of a collateral *trans*-cleavage of the quenched-fluorescent ssDNA/ssRNA by Cas12/Cas13, respectively. (II) CRISPR-Cas9 enrichment for NGS detection (Quan et al., 2019). (a) DNA or cDNA sequences containing the gene of interest (green); (b) blockage of DNA extremities; (c) CRISPR-Cas9 cleaves the target gene into fragments appropriately sized for NGS (yellow arrows); (d) ligation of universal sequencing adapters; (e) enrichment of the target sequence, which is now ready for NGS.

The collateral effect of Cas12 and Cas13 has been used as a key step to create several diagnostic platforms, such as DETECTR, HOLMES (both using Cas12), SHERLOCK, and CARMEN-Cas13 (the last two using Cas13) (Gootenberg et al., 2017; Chen et al., 2018; Li S. Y. et al., 2018; Ackerman et al., 2020). All platforms have similar diagnostic strategies, which involve the incubation of Cas enzymes (Cas12/Cas13) along with the target nucleic acid and fluorescent ssDNA/ssRNA reporters. By detecting the target nucleic acid, the Cas enzymes *trans-*cleave the quenched-fluorescent ssDNA/ssRNA reporters inserted into the platform, generating a robust fluorescent signal from around 1-h incubation (Figure 3) with a good correlation with PCR-based methods (Gootenberg et al., 2017; Chen et al., 2018; Gootenberg et al., 2018). In order to achieve attomolar sensitivity, the nucleic acid detection platforms were coupled to DNA amplification steps (i.e., PCR, recombinase polymerase amplification, and loop-mediated amplification) or reverse transcriptase combined with a DNA amplification step and T7 transcription for RNA targets (Gootenberg et al., 2017; Chen et al., 2018; Gootenberg et al., 2018; Li S. Y. et al., 2018; Ackerman et al., 2020; Broughton et al., 2020). To further enhance signal sensitivity, CRISPR type III effector nuclease Csm6, responsible for non-specific RNA degradation, can be combined with Cas13 activity (Gootenberg et al., 2018).

CARMEN-Cas13 and SHERLOCK have also been explored for multiplexing assays. The first one was specifically developed for this purpose and uses droplets containing either sample or detection solution, arranged pairwise. CARMEN can test more than 4500 crRNA–target pairs on a single microfluidic chip, which represents a simultaneous detection of around 170 agents (Ackerman et al., 2020). SHERLOCK, however, explored different *trans*-cleavage ssRNA preferences of Cas13 orthologs to develop a four-channel single-reaction multiplexing (Gootenberg et al., 2018). A different multiplexing strategy is employed by FLASH, a platform that uses Cas9 to enrich low-abundance targets from complex backgrounds (including clinical specimens) before NGS (Figure 3; Quan et al., 2019). Both CARMEN-Cas13 and FLASH offered an important multiplexing capacity but rely on robust laboratory structure, which may impar its implementation in less developed regions. SHERLOCK, however, has demonstrated its feasibility also as a paper-based test, which amplifies its potential to become a widely spread quick-and-cheap ($0.61 per test) diagnostic method (Gootenberg et al., 2017). The addition of an extra step called HUDSON before SHERLOCK protocol enabled the viral detection directly from bodily fluids, contributing to the creation of a field-deployable diagnostic platform (Myhrvold et al., 2018). Its efficiency to detect bacteria directly from patient samples, however, is yet to be defined. Also, SHERLOCK might benefit from Cas13 engineering in order to increase target preference options and therefore the multiplexing panel.

When focusing on ARGs, *Klebsiella pneumoniae* carbapenemase (KPC) and New Delhi metallo-β-lactamase (NDM) were precisely detected and distinguished from five clinical isolates of *K. pneumoniae* (Gootenberg et al., 2017). Also, HIV drug resistance mutations from 22 patient samples could be identified (Ackerman et al., 2020). As an advantage, these platforms offer a highly specific detection of single-nucleotide polymorphisms (SNPs), which can be valuable to precisely distinguish any desired resistance gene variant (Li S. Y. et al., 2018; Myhrvold et al., 2018).

Of note, the power of the platforms to face real-world challenges has been demonstrated with the detection of SARS-CoV-2 during the COVID-19 pandemic (Ackerman et al., 2020; Broughton et al., 2020) and with the FDA emergency authorization for CRISPR SARS-CoV-2 Rapid Diagnostic using SHERLOCK platform (Guglielmi, 2020).

When employed against AMR, CRISPR-Cas9, Cas3, and Cas13 have been explored as a powerful sequence-specific antimicrobial. Cell death is an expected outcome when sgRNA is directed to genes on the chromosome or plasmids containing a toxin–antitoxin system. For vectors without toxin–antitoxin, plasmid clearance or drastic copy number reduction can be achieved when targeting plasmids up to 300 copies/cell (Bikard et al., 2014; Citorik et al., 2014; Yosef et al., 2015; Kiga et al., 2020; Tagliaferri et al., 2020). Consequently to plasmid clearance in clinical isolates, other non-targeting ARGs harbored on the target vector were also eliminated, and antibiotic reusability could be confirmed in a *Galleria mellonella* infection model (Tagliaferri et al., 2020). Several variants or sub-variants of the resistance gene can be covered with one sgRNA/crRNA directed to a conserved genetic region (Kim et al., 2016; Tagliaferri et al., 2020), while selecting a sequence from a variable region may be a strategy to achieve a narrow-spectrum effect. CRISPR-Cas-mediated interference can also be fine-tuned according to the delivery approach. CRISPR-Cas delivery can be mediated by bacteriophages, and the specificity of phage–host interactions is an advantage when the target is placed in complex environments, such as microbiota (Bikard et al., 2014; Citorik et al., 2014; Yosef et al., 2015). Alternatively, the CRISPR-Cas system can be delivered via conjugative plasmid (Citorik et al., 2014; Rodrigues et al., 2019; Ruotsalainen et al., 2019). Opposite to the phage-mediated approach, lack of specific receptors for plasmid uptake during conjugation is considered as an advantage over phage delivery, as mutations in the receptors may lead to phage resistance. On the other hand, the conjugation rate is slower when compared to transduction (Ruotsalainen et al., 2019).

## Discussion and Future Directions

Even with eminent demand, little has been explored of CRISPR-Cas and aptamer potential toward treatment of bacterial infection. As for CRISPR-Cas approach, Cas9 immunogenicity must be considered (Crudele and Chamberlain, 2018), as well as the definition of the most appropriate delivery method to optimize CRISPR-Cas effect in targeting bacteria within complex microbial communities. Environmental assessments may be required to evaluate risks involved on plasmid clearance and bacterial death, which can affect the frequency of non-targeting bacterial species and non-targeting plasmids. Also, with the ordinary or induced death of the targeting bacteria, CRISPR-Cas nucleic acid will naturally be released into the environment and strategies to prevent spread and horizontal transference of CRISPR-Cas system still need to be developed.

Important limitations of *in vivo* use of aptamers stem from their susceptibility to nuclease degradation and rapid elimination due to renal filtration, but chemical adjustments to the oligonucleotide structure have contributed to decrease those shortcomings (Rohloff et al., 2014). As an FDA-approved aptamer-based therapy is already a reality, we believe that the extension of this technology to other fields, including AMR, is a matter of time.

In contrast to treatment application, CRISPR-Cas-mediated diagnostic has been recently FDA-approved for detecting SARS-CoV-2, paving the way for further applications. Its high scalability and multiplexing properties are of great value for the detection and surveillance of the wide varieties of ARGs. A limitation of this approach is the target of either DNA or RNA, which confirms the presence but not the functionality of the ARGs. Aptamers by their turn target ARG products, but in order to be used as an independent diagnostic tool, increased sensitivity to attomolar levels may be required to bacterial detection in bloodstream (Kelley, 2017). As a counterpoint, the target flexibility of CRISPR-Cas, its simplicity, and the rational design of sgRNA/crRNA can be an advantage over the more complex and randomized process of aptamer selection.

We envisioned that, in the near future, CRISPR-Cas and aptamers can be combined to treat and/or diagnose resistant bacterial infections due to their aforementioned complementary characteristics. Together, those strategies have already shown to reduce CRISPR-Cas-related off-target effects in the HEK293 cell line (Zhao et al., 2020) and to increase the delivery selectivity in liver cells (Zhuang et al., 2020), and the combination was a powerful and reliable molecular sensor able to detect nasopharyngeal carcinoma biomarkers (Li et al., 2021). Whether their combined characteristics will also be beneficial for AMR diagnostics and for treating infections caused by resistant bacteria is yet to be determined. A recent study developed a strategy to recognize surface proteins on MRSA strains by aptamer and CRISPR-Cas12a-assisted rolling circle amplification (Xu et al., 2020). Still, there is a gallery of CRISPR-Cas/aptamer combinations and target bacteria to be tested, as well as further optimizations to achieve attomolar sensitivity.

Studies employing aptamer and CRISPR-Cas for diagnostics have demonstrated their ability to provide shorter turnaround time results than the gold standard AMR phenotypic tests, which can take up to 72 h to be released. This, along with the possibility of developing paper-based diagnostics, highlights the techniques’ potential to be employed as a first guidance to clinical decisions related to antimicrobial use. Altogether, the molecular approaches may offer a suitable solution to circumvent antibiotic misuse in the first antibiotic prescription, currently guided only by empirical decisions.

## Author Contributions

HP, TT, and TM designed and wrote the manuscript. HP and TT analyzed the data. All authors contributed to the article and approved the submitted version.

## Conflict of Interest

The authors declare that the research was conducted in the absence of any commercial or financial relationships that could be construed as a potential conflict of interest.

## Footnotes

1 https://covid19.who.int/

## Abbreviations

ALISA: Aptamer-linked immobilized sorbent assay
AMR: Antimicrobial resistance
ARG: Antimicrobial resistance genes
CRISPR-Cas: Clustered regularly interspaced short palindromic repeats, CRISPR-associated enzymes
crRNA: CRISPR RNA
COVID-19: Coronavirus disease 2019
DNA: Deoxyribonucleic acid
FDA: Food and Drug Administration
HIV: Human immunodeficiency virus
KPC: *Klebsiella pneumoniae* carbapenemase
LAMP: Loop-mediated isothermal amplification
MALDI-TOF MS: Matrix-assisted laser desorption/ionization time-of-flight mass spectrometry
MRSA: Methicillin-resistant *Staphylococcus aureus*
NDM: New Delhi metallo- β-lactamase
NGS: Next-generation sequencing
PAM: Protospacer adjacent motif
PBP: Penicillin-binding protein
RNA: Ribonucleic acid
RNP: Ribonucleoprotein
RT-qPCR: quantitative reverse transcription PCR
SARS: Severe acute respiratory syndrome
SARS-CoV-2: Severe acute respiratory syndrome coronavirus 2
SELEX: Systematic evolution of ligands by exponential enrichment
sgRNA: single-guide RNA
siRNA: Small interfering RNA
SNP: Single-nucleotide polymorphism
TALEN: Transcription activator-like effector nucleases
tracrRNA: trans-activating CRISPR RNA.

## References

[B1] Abudayyeh O. O. Gootenberg J. S. Konermann S. Joung J. Slaymaker I. M. Cox D. B. (2016). C2c2 is a single-component programmable RNA-guided RNA-targeting CRISPR effector. *Science* 353:aaf5573. 10.1126/science.aaf5573 27256883 PMC5127784

[B2] Ackerman C. M. Myhrvold C. Thakku S. G. Freije C. A. Metsky H. C. Yang D. K. (2020). Massively multiplexed nucleic acid detection using Cas13. *Nature* 582 277–282. 10.1038/s41586-020-2279-8 32349121 PMC7332423

[B3] Adams C. P. Brantner V. V. (2010). Spending on new drug development1. *Health Econ.* 19 130–141. 10.1002/hec.1454 19247981

[B4] Afrasiabi S. Pourhajibagher M. Raoofian R. Tabarzad M. Bahador A. (2020). Therapeutic applications of nucleic acid aptamers in microbial infections. *J. Biomed. Sci.* 27:6. 10.1186/s12929-019-0611-0 PMC6941257 31900238

[B5] Ahn J. Y. Lee K. A. Lee M. J. Sekhon S. S. Rhee S. K. Cho S. J. (2018). Surface plasmon resonance aptamer biosensor for discriminating pathogenic bacteria vibrio parahaemolyticus. *J. Nanosci. Nanotechnol.* 18 1599–1605. 10.1166/jnn.2018.14212 29448635

[B6] Alfavian H. Mousavi Gargari S. L. Rasoulinejad S. Medhat A. (2017). Development of a DNA aptamer that binds specifically to group a streptococcus serotype M3. *Can. J. Microbiol.* 63 160–168. 10.1139/cjm-2016-0495 28121169

[B7] Allen H. K. Donato J. Wang H. H. Cloud-Hansen K. A. Davies J. Handelsman J. (2010). Call of the wild: antibiotic resistance genes in natural environments. *Nat. Rev. Microbiol.* 8 251–259. 10.1038/nrmicro2312 20190823

[B8] Amraee M. Oloomi M. Yavari A. Bouzari S. (2017). DNA aptamer identification and characterization for *E. coli* O157 detection using cell based SELEX method. *Anal. Biochem.* 536 36–44. 10.1016/j.ab.2017.08.005 28818557

[B9] Appaturi J. N. Pulingam T. Thong K. L. Muniandy S. Ahmad N. Leo B. F. (2020). Rapid and sensitive detection of Salmonella with reduced graphene oxide-carbon nanotube based electrochemical aptasensor. *Anal. Biochem.* 589:113489. 10.1016/j.ab.2019.113489 31655050

[B10] Bai L. Chen Y. Bai Y. Chen Y. Zhou J. Huang A. (2017). Fullerene-doped polyaniline as new redox nanoprobe and catalyst in electrochemical aptasensor for ultrasensitive detection of *Mycobacterium tuberculosis* MPT64 antigen in human serum. *Biomaterials* 133 11–19. 10.1016/j.biomaterials.2017.04.010 28414975

[B11] Bayrac C. Oktem H. A. (2017). Evaluation of *Staphylococcus aureus* DNA aptamer by enzyme-linked aptamer assay and isothermal titration calorimetry. *J. Mol. Recognit.* 30 1–9. 10.1002/jmr.2583 27696554

[B12] Bayrac C. Eyidogan F. Avni Oktem H. (2017). DNA aptamer-based colorimetric detection platform for *Salmonella Enteritidis*. *Biosens. Bioelectron.* 98 22–28. 10.1016/j.bios.2017.06.029 28646719

[B13] Bikard D. Euler C. W. Jiang W. Nussenzweig P. M. Goldberg G. W. Duportet X. (2014). Exploiting CRISPR-Cas nucleases to produce sequence-specific antimicrobials. *Nat. Biotechnol.* 32 1146–1150. 10.1038/nbt.3043 25282355 PMC4317352

[B14] Blair J. M. Webber M. A. Baylay A. J. Ogbolu D. O. Piddock L. J. (2015). Molecular mechanisms of antibiotic resistance. *Nat. Rev. Microbiol.* 13 42–51. 25435309 10.1038/nrmicro3380

[B15] Broughton J. P. Deng X. Yu G. Fasching C. L. Servellita V. Singh J. (2020). CRISPR-Cas12-based detection of SARS-CoV-2. *Nat Biotechnol.* 38 870–874. 32300245 10.1038/s41587-020-0513-4 PMC9107629

[B16] Bush K. Bradford P. A. (2019). Interplay between beta-lactamases and new beta-lactamase inhibitors. *Nat. Rev. Microbiol.* 17 295–306. 10.1038/s41579-019-0159-8 30837684

[B17] Cai R. Yin F. Zhang Z. Tian Y. Zhou N. (2019). Functional chimera aptamer and molecular beacon based fluorescent detection of *Staphylococcus aureus* with strand displacement-target recycling amplification. *Anal. Chim. Acta* 1075 128–136. 10.1016/j.aca.2019.05.014 31196418

[B18] Calero-Caceres W. Ye M. Balcazar J. L. (2019). Bacteriophages as environmental reservoirs of antibiotic resistance. *Trends Microbiol.* 27 570–577. 10.1016/j.tim.2019.02.008 30905524

[B19] Cao L. Gao C. H. Zhu J. Zhao L. Wu Q. Li M. (2016). Identification and functional study of type III-A CRISPR-Cas systems in clinical isolates of *Staphylococcus aureus*. *Int. J. Med. Microbiol.* 306 686–696. 10.1016/j.ijmm.2016.08.005 27600408

[B20] Casini A. Olivieri M. Petris G. Montagna C. Reginato G. Maule G. (2018). A highly specific SpCas9 variant is identified by in vivo screening in yeast. *Nat. Biotechnol.* 36 265–271. 10.1038/nbt.4066 29431739 PMC6066108

[B21] CDC, (2019). *Antibiotic Resistance Threats in the United States.* Atlanta: CDC.

[B22] Chen J. S. Dagdas Y. S. Kleinstiver B. P. Welch M. M. Sousa A. A. Harrington L. B. (2017). Enhanced proofreading governs CRISPR-Cas9 targeting accuracy. *Nature* 550 407–410. 10.1038/nature24268 28931002 PMC5918688

[B23] Chen J. S. Ma E. Harrington L. B. Da Costa M. Tian X. Palefsky J. M. (2018). CRISPR-Cas12a target binding unleashes indiscriminate single-stranded DNase activity. *Science* 360 436–439. 10.1126/science.aar6245 29449511 PMC6628903

[B24] Chen K. Gao C. (2014). Targeted genome modification technologies and their applications in crop improvements. *Plant Cell Rep.* 33 575–583. 10.1007/s00299-013-1539-6 24277082

[B25] Chen L. Rashid F. Shah A. Awan H. M. Wu M. Liu A. (2015). The isolation of an RNA aptamer targeting to p53 protein with single amino acid mutation. *Proc. Natl. Acad. Sci. U.S.A.* 112 10002–10007. 10.1073/pnas.1502159112 26216949 PMC4538674

[B26] Chinnappan R. Alamer S. Eissa S. Rahamn A. A. Abu Salah K. M. Zourob M. (2017). Fluorometric graphene oxide-based detection of *Salmonella enteritis* using a truncated DNA aptamer. *Mikrochim. Acta* 185:61. 10.1007/s00604-017-2601-9 29594712

[B27] Citorik R. J. Mimee M. Lu T. K. (2014). Sequence-specific antimicrobials using efficiently delivered RNA-guided nucleases. *Nat. Biotechnol.* 32 1141–1145. 10.1038/nbt.3011 25240928 PMC4237163

[B28] Crudele J. M. Chamberlain J. S. (2018). Cas9 immunity creates challenges for CRISPR gene editing therapies. *Nat. Commun.* 9:3497. 10.1038/s41467-018-05843-9 PMC6115392 30158648

[B29] Dalirirad S. Steckl A. J. (2020). Lateral flow assay using aptamer-based sensing for on-site detection of dopamine in urine. *Anal. Biochem.* 596:113637. 10.1016/j.ab.2020.113637 32087129

[B30] Das R. Dhiman A. Kapil A. Bansal V. Sharma T. K. (2019a). Aptamer-mediated colorimetric and electrochemical detection of *Pseudomonas aeruginosa* utilizing peroxidase-mimic activity of gold NanoZyme. *Anal. Bioanal. Chem.* 411 1229–1238. 10.1007/s00216-018-1555-z 30637436

[B31] Das R. Dhiman A. Mishra S. K. Haldar S. Sharma N. Bansal A. (2019b). Structural switching electrochemical DNA aptasensor for the rapid diagnosis of tuberculous meningitis. *Int. J. Nanomed.* 14 2103–2113. 10.2147/ijn.s189127 30988611 PMC6440448

[B32] D’costa V. M. King C. E. Kalan L. Morar M. Sung W. W. Schwarz C. (2011). Antibiotic resistance is ancient. *Nature* 477 457–461. 21881561 10.1038/nature10388

[B33] Dehghani Z. Hosseini M. Mohammadnejad J. Bakhshi B. Rezayan A. H. (2018). Colorimetric aptasensor for *Campylobacter jejuni* cells by exploiting the peroxidase like activity of Au@Pd nanoparticles. *Mikrochim. Acta* 185:448. 10.1007/s00604-018-2976-2 30187142

[B34] Dekker J. P. (2018). Metagenomics for clinical infectious disease diagnostics steps closer to reality. *J. Clin. Microbiol.* 56:e00850-18. 10.1128/JCM.00850-18 PMC6113477 29976592

[B35] Doi Y. Wachino J. I. Arakawa Y. (2016). Aminoglycoside resistance: the emergence of acquired 16S ribosomal RNA methyltransferases. *Infect. Dis. Clin. North Am.* 30 523–537. 27208771 10.1016/j.idc.2016.02.011 PMC4878400

[B36] Dua P. Ren S. Lee S. W. Kim J. K. Shin H. S. Jeong O. C. (2016). Cell-SELEX based identification of an RNA aptamer for *Escherichia coli* and its use in various detection formats. *Mol. Cells* 39 807–813. 10.14348/molcells.2016.0167 27871171 PMC5125936

[B37] Duan N. Xu B. Wu S. Wang Z. (2016). Magnetic nanoparticles-based aptasensor using gold nanoparticles as colorimetric probes for the detection of *Salmonella typhimurium*. *Anal. Sci.* 32 431–436. 10.2116/analsci.32.431 27063716

[B38] Ellington A. D. Szostak J. W. (1990). In vitro selection of RNA molecules that bind specific ligands. *Nature* 346 818–822. 10.1038/346818a0 1697402

[B39] Esposito C. L. Nuzzo S. Catuogno S. Romano S. De Nigris F. De Franciscis V. (2018). STAT3 gene silencing by aptamer-siRNA chimera as selective therapeutic for glioblastoma. *Mol. Ther. Nucleic Acids* 10 398–411. 10.1016/j.omtn.2017.12.021 29499951 PMC5862137

[B40] Fan Y. Cui M. Liu Y. Jin M. Zhao H. (2020). Selection and characterization of DNA aptamers for constructing colorimetric biosensor for detection of PBP2a. *Spectrochim. Acta A Mol. Biomol. Spectrosc.* 228:117735. 10.1016/j.saa.2019.117735 31757698

[B41] Fischer J. E. Harbarth S. Agthe A. G. Benn A. Ringer S. A. Goldmann D. A. (2004). Quantifying uncertainty: physicians’ estimates of infection in critically ill neonates and children. *Clin. Infect. Dis.* 38 1383–1390. 10.1086/420741 15156475

[B42] Gao R. Zhong Z. Gao X. Jia L. (2018). Graphene oxide quantum dots assisted construction of fluorescent aptasensor for rapid detection of *Pseudomonas aeruginosa* in food samples. *J. Agric. Food Chem.* 66 10898–10905. 10.1021/acs.jafc.8b02164 30247907

[B43] Gleditzsch D. Pausch P. Muller-Esparza H. Ozcan A. Guo X. Bange G. (2019). PAM identification by CRISPR-Cas effector complexes: diversified mechanisms and structures. *RNA Biol.* 16 504–517. 10.1080/15476286.2018.1504546 30109815 PMC6546366

[B44] Gootenberg J. S. Abudayyeh O. O. Kellner M. J. Joung J. Collins J. J. Zhang F. (2018). Multiplexed and portable nucleic acid detection platform with Cas13. Cas12a, and Csm6. *Science* 360 439–444. 10.1126/science.aaq0179 29449508 PMC5961727

[B45] Gootenberg J. S. Abudayyeh O. O. Lee J. W. Essletzbichler P. Dy A. J. Joung J. (2017). Nucleic acid detection with CRISPR-Cas13a/C2c2. *Science* 356 438–442. 10.1126/science.aam9321 28408723 PMC5526198

[B46] Gragoudas E. S. Adamis A. P. Cunningham E. T. Jr. Feinsod M. Guyer D. R. (2004). Pegaptanib for neovascular age-related macular degeneration. *N. Engl. J. Med.* 351 2805–2816. 15625332 10.1056/NEJMoa042760

[B47] Guglielmi G. (2020). First CRISPR test for the coronavirus approved in the United States. *Nature* 10.1038/d41586-020-01402-9 Online ahead of print. 32385368

[B48] Ha N. R. Jung I. P. La I. J. Jung H. S. Yoon M. Y. (2017). Ultra-sensitive detection of kanamycin for food safety using a reduced graphene oxide-based fluorescent aptasensor. *Sci. Rep.* 7:40305. 10.1038/srep40305 PMC5215691 28054670

[B49] Hamula C. L. Peng H. Wang Z. Tyrrell G. J. Li X. F. Le X. C. (2016). An improved SELEX technique for selection of DNA aptamers binding to M-type 11 of *Streptococcus pyogenes*. *Methods* 97 51–57. 10.1016/j.ymeth.2015.12.005 26678795

[B50] Hille F. Richter H. Wong S. P. Bratovic M. Ressel S. Charpentier E. (2018). The biology of CRISPR-Cas: backward and forward. *Cell* 172 1239–1259. 10.1016/j.cell.2017.11.032 29522745

[B51] Hua R. Hao N. Lu J. Qian J. Liu Q. Li H. (2018). A sensitive Potentiometric resolved ratiometric Photoelectrochemical aptasensor for *Escherichia coli* detection fabricated with non-metallic nanomaterials. *Biosens. Bioelectron.* 106 57–63. 10.1016/j.bios.2018.01.053 29414089

[B52] Jin B. Wang S. Lin M. Jin Y. Zhang S. Cui X. (2017). Upconversion nanoparticles based FRET aptasensor for rapid and ultrasenstive bacteria detection. *Biosens. Bioelectron.* 90 525–533. 10.1016/j.bios.2016.10.029 27825886

[B53] Jinek M. Chylinski K. Fonfara I. Hauer M. Doudna J. A. Charpentier E. (2012). A programmable dual-RNA-guided DNA endonuclease in adaptive bacterial immunity. *Science* 337 816–821. 10.1126/science.1225829 22745249 PMC6286148

[B54] Kaur H. Bruno J. G. Kumar A. Sharma T. K. (2018). Aptamers in the therapeutics and diagnostics pipelines. *Theranostics* 8 4016–4032. 10.7150/thno.25958 30128033 PMC6096388

[B55] Kelley S. O. (2017). What are clinically relevant levels of cellular and biomolecular analytes? *ACS Sens.* 2 193–197. 10.1021/acssensors.6b00691 28723142

[B56] Kiga K. Tan X. E. Ibarra-Chavez R. Watanabe S. Aiba Y. Sato’o Y. (2020). Development of CRISPR-Cas13a-based antimicrobials capable of sequence-specific killing of target bacteria. *Nat. Commun.* 11:2934. 10.1038/s41467-020-16731-6 PMC7287087 32523110

[B57] Kim H. J. Park J. Y. Lee T. S. Song I. H. Cho Y. L. Chae J. R. (2019). PET imaging of HER2 expression with an 18F-fluoride labeled aptamer. *PLoS One* 14:e0211047. 10.1371/journal.pone.0211047 30682091 PMC6347211

[B58] Kim J. S. Cho D. H. Park M. Chung W. J. Shin D. Ko K. S. (2016). CRISPR/Cas9-mediated re-sensitization of antibiotic-resistant *Escherichia coli* harboring extended-spectrum beta-lactamases. *J. Microbiol. Biotechnol.* 26 394–401. 10.4014/jmb.1508.08080 26502735

[B59] Kleinstiver B. P. Pattanayak V. Prew M. S. Tsai S. Q. Nguyen N. T. Zheng Z. (2016). High-fidelity CRISPR-Cas9 nucleases with no detectable genome-wide off-target effects. *Nature* 529 490–495. 10.1038/nature16526 26735016 PMC4851738

[B60] Kurt H. Yuce M. Hussain B. Budak H. (2016). Dual-excitation upconverting nanoparticle and quantum dot aptasensor for multiplexed food pathogen detection. *Biosens. Bioelectron.* 81 280–286. 10.1016/j.bios.2016.03.005 26971274

[B61] Lavu P. S. Mondal B. Ramlal S. Murali H. S. Batra H. V. (2016). Selection and characterization of aptamers using a modified whole cell bacterium SELEX for the detection of *Salmonella enterica* serovar Typhimurium. *ACS Comb. Sci.* 18 292–301. 10.1021/acscombsci.5b00123 27070414

[B62] Lee J. K. Jeong E. Lee J. Jung M. Shin E. Kim Y. H. (2018). Directed evolution of CRISPR-Cas9 to increase its specificity. *Nat. Commun.* 9:3048. 10.1038/s41467-018-05477-x PMC6078992 30082838

[B63] Leekha S. Terrell C. L. Edson R. S. (2011). General principles of antimicrobial therapy. *Mayo Clin. Proc.* 86 156–167. 10.4065/mcp.2010.0639 21282489 PMC3031442

[B64] Li H. Xing S. Xu J. He Y. Lai Y. Wang Y. (2021). Aptamer-based CRISPR/Cas12a assay for the ultrasensitive detection of extracellular vesicle proteins. *Talanta* 221:121670. 10.1016/j.talanta.2020.121670 33076176

[B65] Li H. Yang Y. Hong W. Huang M. Wu M. Zhao X. (2020). Applications of genome editing technology in the targeted therapy of human diseases: mechanisms, advances and prospects. *Signal Transduct. Target. Ther.* 5:1. 10.1038/s41392-019-0089-y PMC6946647 32296011

[B66] Li N. Huang X. Sun D. Yu W. Tan W. Luo Z. (2018). Dual-aptamer-based voltammetric biosensor for the *Mycobacterium tuberculosis* antigen MPT64 by using a gold electrode modified with a peroxidase loaded composite consisting of gold nanoparticles and a Zr(IV)/terephthalate metal-organic framework. *Mikrochim. Acta* 185:543. 10.1007/s00604-018-3081-2 30421038

[B67] Li S. Y. Cheng Q. X. Wang J. M. Li X. Y. Zhang Z. L. Gao S. (2018). CRISPR-Cas12a-assisted nucleic acid detection. *Cell Discov.* 4:20. 10.1038/s41421-018-0028-z PMC5913299 29707234

[B68] Liu H. L. C. Zhao Y. Han X. Zhou Z. Wang C. Li R. (2018). Comparing successful gene knock-in efficiencies of CRISPR/Cas9 with ZFNs and TALENs gene editing systems in bovine and dairy goat fetal fibroblasts. *J. Integr. Agric.* 17 406–414. 10.1016/s2095-3119(17)61748-9

[B69] Liu L. Li X. Ma J. Li Z. You L. Wang J. (2017). The molecular architecture for RNA-guided RNA cleavage by cas13a. *Cell* 170:e710. 10.1016/j.cell.2017.06.050 28757251

[B70] Liu Y. Jiang W. Yang S. Hu J. Lu H. Han W. (2019). Rapid detection of mycoplasma-infected cells by an ssDNA aptamer probe. *ACS Sens.* 4 2028–2038. 10.1021/acssensors.9b00582 31403764

[B71] Llor C. Bjerrum L. (2014). Antimicrobial resistance: risk associated with antibiotic overuse and initiatives to reduce the problem. *Ther. Adv. Drug Saf.* 5 229–241. 10.1177/2042098614554919 25436105 PMC4232501

[B72] Makarova K. S. Wolf Y. I. Alkhnbashi O. S. Costa F. Shah S. A. Saunders S. J. (2015). An updated evolutionary classification of CRISPR-Cas systems. *Nat. Rev. Microbiol.* 13 722–736. 26411297 10.1038/nrmicro3569 PMC5426118

[B73] Makarova K. S. Wolf Y. I. Iranzo J. Shmakov S. A. Alkhnbashi O. S. Brouns S. J. J. (2020). Evolutionary classification of CRISPR-Cas systems: a burst of class 2 and derived variants. *Nat. Rev. Microbiol.* 18 67–83. 10.1038/s41579-019-0299-x 31857715 PMC8905525

[B74] Maldonado J. Estevez M. C. Fernandez-Gavela A. Gonzalez-Lopez J. J. Gonzalez-Guerrero A. B. Lechuga L. M. (2020). Label-free detection of nosocomial bacteria using a nanophotonic interferometric biosensor. *Analyst* 145 497–506. 10.1039/c9an01485c 31750459

[B75] Malik B. Bhattacharyya S. (2019). Antibiotic drug-resistance as a complex system driven by socio-economic growth and antibiotic misuse. *Sci. Rep.* 9:9788. 10.1038/s41598-019-46078-y PMC6611849 31278344

[B76] Marraffini L. A. Sontheimer E. J. (2010). Self versus non-self discrimination during CRISPR RNA-directed immunity. *Nature* 463 568–571. 10.1038/nature08703 20072129 PMC2813891

[B77] Marton S. Cleto F. Krieger M. A. Cardoso J. (2016). Isolation of an Aptamer that Binds Specifically to *E. coli*. *PLoS One* 11:e0153637. 10.1371/journal.pone.0153637 27104834 PMC4841571

[B78] McKeague M. De Girolamo A. Valenzano S. Pascale M. Ruscito A. Velu R. (2015). Comprehensive analytical comparison of strategies used for small molecule aptamer evaluation. *Anal. Chem.* 87 8608–8612. 10.1021/acs.analchem.5b02102 26192270

[B79] Mirzakhani K. Mousavi Gargari S. L. Rasooli I. Rasoulinejad S. (2017). Development of a DNA Aptamer for screening *Neisseria meningitidis* serogroup B by cell selex. *Iran Biomed. J.* 22 193–201. 28941453 10.22034/ibj.22.3.193 PMC5889504

[B80] Mitsakakis K. Kaman W. E. Elshout G. Specht M. Hays J. P. (2018). Challenges in identifying antibiotic resistance targets for point-of-care diagnostics in general practice. *Future Microbiol.* 13 1157–1164. 10.2217/fmb-2018-0084 30113214 PMC6190172

[B81] Mojica F. J. M. Diez-Villasenor C. Garcia-Martinez J. Almendros C. (2009). Short motif sequences determine the targets of the prokaryotic CRISPR defence system. *Microbiology* 155 733–740. 10.1099/mic.0.023960-0 19246744

[B82] Mol A. A. Groher F. Schreiber B. Ruhmkorff C. Suess B. (2019). Robust gene expression control in human cells with a novel universal TetR aptamer splicing module. *Nucleic Acids Res.* 47:e132. 10.1093/nar/gkz753 31504742 PMC6846422

[B83] Mozioglu E. Gokmen O. Tamerler C. Kocagoz Z. T. Akgoz M. (2016). Selection of Nucleic acid aptamers specific for *Mycobacterium tuberculosis*. *Appl. Biochem. Biotechnol.* 178 849–864. 10.1007/s12010-015-1913-7 26541162

[B84] Munita J. M. Arias C. A. (2016). Mechanisms of antibiotic resistance. *Microbiol. Spectr.* 4 1–24. 10.1128/microbiolspec.VMBF-0016-2015 PMC4888801 27227291

[B85] Myhrvold C. Freije C. A. Gootenberg J. S. Abudayyeh O. O. Metsky H. C. Durbin A. F. (2018). Field-deployable viral diagnostics using CRISPR-Cas13. *Science* 360 444–448. 10.1126/science.aas8836 29700266 PMC6197056

[B86] O’Neil J. (2016). *Tackling Drug-Resistant Infections Globally: Final Report and Recommendations [Online].* Available online at: http://amr-review.org (accessed July 19, 2020).

[B87] Ota Y. Furuhashi K. Nanba T. Yamanaka K. Ishikawa J. Nagura O. (2019). A rapid and simple detection method for phenotypic antimicrobial resistance in *Escherichia coli* by loop-mediated isothermal amplification. *J. Med. Microbiol.* 68 169–177. 10.1099/jmm.0.000903 30624176

[B88] Pancholi P. Carroll K. C. Buchan B. W. Chan R. C. Dhiman N. Ford B. (2018). Multicenter evaluation of the accelerate phenotest BC kit for rapid identification and phenotypic antimicrobial susceptibility testing using morphokinetic cellular analysis. *J. Clin. Microbiol.* 56:e01329-17. 10.1128/JCM.01329-17 PMC5869823 29305546

[B89] Patrinos G. P. Danielson P. B. Ansorge W. J. (2017). “Molecular diagnostics: past, present, and future,” in *Molecular Diagnostic* ed. Patrinos G.P., (Amsterdam: Elsevier), 520.

[B90] Peng W. Feng M. Feng X. Liang Y. X. She Q. (2015). An archaeal CRISPR type III-B system exhibiting distinctive RNA targeting features and mediating dual RNA and DNA interference. *Nucleic Acids Res.* 43 406–417. 10.1093/nar/gku1302 25505143 PMC4288192

[B91] Powers J. H. (2004). Antimicrobial drug development–the past, the present, and the future. *Clin. Microbiol. Infect* 10 (Suppl. 4), 23–31. 10.1111/j.1465-0691.2004.1007.x 15522037

[B92] Qiao J. Meng X. Sun Y. Li Q. Zhao R. Zhang Y. (2018). Aptamer-based fluorometric assay for direct identification of methicillin-resistant *Staphylococcus aureus* from clinical samples. *J. Microbiol. Methods* 153 92–98. 10.1016/j.mimet.2018.09.011 30243766

[B93] Quan J. Langelier C. Kuchta A. Batson J. Teyssier N. Lyden A. (2019). FLASH: a next-generation CRISPR diagnostic for multiplexed detection of antimicrobial resistance sequences. *Nucleic Acids Res.* 47:e83. 10.1093/nar/gkz418 31114866 PMC6698650

[B94] Rasoulinejad S. Gargari S. L. M. (2016). Aptamer-nanobody based ELASA for specific detection of *Acinetobacter baumannii* isolates. *J. Biotechnol.* 231 46–54. 10.1016/j.jbiotec.2016.05.024 27234880

[B95] Reich P. Stoltenburg R. Strehlitz B. Frense D. Beckmann D. (2017). Development of an impedimetric aptasensor for the detection of *Staphylococcus aureus*. *Int. J. Mol. Sci.* 18:2484. 10.3390/ijms18112484 29160851 PMC5713450

[B96] Ren J. Liang G. Man Y. Li A. Jin X. Liu Q. (2019). Aptamer-based fluorometric determination of *Salmonella Typhimurium* using Fe3O4 magnetic separation and CdTe quantum dots. *PLoS One* 14:e0218325. 10.1371/journal.pone.0218325 31216306 PMC6584018

[B97] Rodrigues M. Mcbride S. W. Hullahalli K. Palmer K. L. Duerkop B. A. (2019). Conjugative delivery of CRISPR-Cas9 for the selective depletion of antibiotic-resistant enterococci. *Antimicrob. Agents Chemother.* 63:e01454-9. 10.1128/AAC.01454-19 PMC6811441 31527030

[B98] Rohloff J. C. Gelinas A. D. Jarvis T. C. Ochsner U. A. Schneider D. J. Gold L. (2014). Nucleic acid ligands with protein-like side chains: modified aptamers and their use as diagnostic and therapeutic agents. *Mol. Ther. Nucleic Acids* 3:e201. 10.1038/mtna.2014.49 25291143 PMC4217074

[B99] Ruotsalainen P. Penttinen R. Mattila S. Jalasvuori M. (2019). Midbiotics: conjugative plasmids for genetic engineering of natural gut flora. *Gut Microbes* 10 643–653. 10.1080/19490976.2019.1591136 30951393 PMC6866695

[B100] Sakyi S. A. Aboagye S. Y. Otchere I. D. Liao A. M. Caltagirone T. G. Yeboah-Manu D. (2016). RNA aptamer that specifically binds to mycolactone and serves as a diagnostic tool for diagnosis of buruli ulcer. *PLoS Negl. Trop Dis.* 10:e0004950. 10.1371/journal.pntd.0004950 27776120 PMC5077154

[B101] Semenova E. Jore M. M. Datsenko K. A. Semenova A. Westra E. R. Wanner B. (2011). Interference by clustered regularly interspaced short palindromic repeat (CRISPR) RNA is governed by a seed sequence. *Proc. Natl. Acad. Sci. U.S.A.* 108 10098–10103. 21646539 10.1073/pnas.1104144108 PMC3121866

[B102] Shen H. Wang J. Liu H. Li Z. Jiang F. Wang F. B. (2016). Rapid and selective detection of pathogenic bacteria in bloodstream infections with aptamer-based recognition. *ACS Appl. Mater Interfaces* 8 19371–19378. 10.1021/acsami.6b06671 27411775

[B103] Sheridan C. (2020). Coronavirus and the race to distribute reliable diagnostics. *Nat. Biotechnol.* 38 382–384. 10.1038/d41587-020-00002-2 32265548

[B104] Shin H. S. Gedi V. Kim J. K. Lee D. K. (2019). Detection of gram-negative bacterial outer membrane vesicles using DNA aptamers. *Sci. Rep.* 9:13167. 10.1038/s41598-019-49755-0 PMC6739373 31511614

[B105] Shin W. R. Sekhon S. S. Rhee S. K. Ko J. H. Ahn J. Y. Min J. (2018). Aptamer-based paper strip sensor for detecting vibrio fischeri. *ACS Comb Sci.* 20 261–268. 10.1021/acscombsci.7b00190 29553704

[B106] Singh R. K. Dhama K. Karthik K. Tiwari R. Khandia R. Munjal A. (2017). Advances in diagnosis, surveillance, and monitoring of Zika virus: an update. *Front. Microbiol.* 8:2677. 10.3389/fmicb.2017.02677 PMC5780406 29403448

[B107] Slaymaker I. M. Gao L. Zetsche B. Scott D. A. Yan W. X. Zhang F. (2016). Rationally engineered Cas9 nucleases with improved specificity. *Science* 351 84–88. 10.1126/science.aad5227 26628643 PMC4714946

[B108] Song M. S. Sekhon S. S. Shin W. R. Kim H. C. Min J. Ahn J. Y. (2017). Detecting and discriminating Shigella sonnei using an aptamer-based fluorescent biosensor platform. *Molecules* 22:825. 10.3390/molecules22050825 28513559 PMC6154610

[B109] Song M. Y. Nguyen D. Hong S. W. Kim B. C. (2017). Broadly reactive aptamers targeting bacteria belonging to different genera using a sequential toggle cell-SELEX. *Sci. Rep.* 7:43641. 10.1038/srep43641 PMC5341558 28272554

[B110] Stoltenburg R. Krafcikova P. Viglasky V. Strehlitz B. (2016). G-quadruplex aptamer targeting protein a and its capability to detect *Staphylococcus aureus* demonstrated by Elona. *Sci. Rep.* 6:33812. 10.1038/srep33812 PMC5030626 27650576

[B111] Su Y. Shao C. Huang X. Qi J. Ge R. Guan H. (2018). Extraction and detection of bisphenol a in human serum and urine by aptamer-functionalized magnetic nanoparticles. *Anal. Bioanal. Chem.* 410 1885–1891. 10.1007/s00216-017-0801-0 29372273

[B112] Suh S. H. Choi S. J. Dwivedi H. P. Moore M. D. Escudero-Abarca B. I. Jaykus L. A. (2018). Use of DNA aptamer for sandwich type detection of *Listeria monocytogenes*. *Anal. Biochem.* 557 27–33. 10.1016/j.ab.2018.04.009 29649475

[B113] Sun Y. Duan N. Ma P. Liang Y. Zhu X. Wang Z. (2019). Colorimetric aptasensor based on truncated aptamer and trivalent DNAzyme for *Vibrio parahemolyticus* determination. *J. Agric. Food Chem.* 67 2313–2320. 10.1021/acs.jafc.8b06893 30721047

[B114] Tagliaferri T. L. Guimaraes N. R. Pereira M. P. M. Vilela L. F. F. Horz H. P. Dos Santos S. G. (2020). Exploring the potential of CRISPR-Cas9 under challenging conditions: facing high-copy plasmids and counteracting beta-lactam resistance in clinical strains of *Enterobacteriaceae*. *Front. Microbiol.* 11:578. 10.3389/fmicb.2020.00578 PMC7203346 32425894

[B115] Tang X. L. Wu S. M. Xie Y. Song N. Guan Q. Yuan C. (2016). Generation and application of ssDNA aptamers against glycolipid antigen ManLAM of *Mycobacterium tuberculosis* for TB diagnosis. *J. Infect.* 72 573–586. 10.1016/j.jinf.2016.01.014 26850356

[B116] Toh S. Y. Citartan M. Gopinath S. C. Tang T. H. (2015). Aptamers as a replacement for antibodies in enzyme-linked immunosorbent assay. *Biosens. Bioelectron.* 64 392–403. 10.1016/j.bios.2014.09.026 25278480

[B117] Tuerk C. Gold L. (1990). Systematic evolution of ligands by exponential enrichment: RNA ligands to bacteriophage T4 DNA polymerase. *Science* 249 505–510. 10.1126/science.2200121 2200121

[B118] Wang L. Huo X. Qi W. Xia Z. Li Y. Lin J. (2020). Rapid and sensitive detection of *Salmonella Typhimurium* using nickel nanowire bridge for electrochemical impedance amplification. *Talanta* 211:120715. 10.1016/j.talanta.2020.120715 32070611

[B119] Waseem H. Jameel S. Ali J. Saleem, Ur Rehman H. Tauseef I. (2019). Contributions and challenges of high throughput qPCR for determining antimicrobial resistance in the environment: a critical review. *Molecules* 24:163. 10.3390/molecules24010163 30609875 PMC6337382

[B120] Wu G. Dai Z. Tang X. Lin Z. Lo P. K. Meyyappan M. (2017). Graphene field-effect transistors for the sensitive and selective detection of *Escherichia coli* Using Pyrene-tagged DNA aptamer. *Adv. Healthc Mater* 6:1700736. 10.1002/adhm.201700736 28795534

[B121] Xiang D. Zheng C. Zhou S. F. Qiao S. Tran P. H. Pu C. (2015). Superior Performance of aptamer in tumor penetration over antibody: implication of aptamer-based theranostics in solid tumors. *Theranostics* 5 1083–1097. 10.7150/thno.11711 26199647 PMC4508498

[B122] Xiong Y. Zhang J. Yang Z. Mou Q. Ma Y. Xiong Y. (2020). Functional DNA regulated CRISPR-Cas12a sensors for point-of-care diagnostics of non-nucleic-acid targets. *J. Am. Chem. Soc.* 142 207–213. 10.1021/jacs.9b09211 31800219 PMC7174832

[B123] Xu L. Dai Q. Shi Z. Liu X. Gao L. Wang Z. (2020). Accurate MRSA identification through dual-functional aptamer and CRISPR-Cas12a assisted rolling circle amplification. *J. Microbiol. Methods* 173:105917. 10.1016/j.mimet.2020.105917 32289369

[B124] Xu Y. Wang H. Luan C. Liu Y. Chen B. Zhao Y. (2018). Aptamer-based hydrogel barcodes for the capture and detection of multiple types of pathogenic bacteria. *Biosens. Bioelectron.* 100 404–410. 10.1016/j.bios.2017.09.032 28957705

[B125] Yoon S. Rossi J. J. (2018). Aptamers: uptake mechanisms and intracellular applications. *Adv. Drug Deliv. Rev.* 134 22–35. 10.1016/j.addr.2018.07.003 29981799 PMC7126894

[B126] Yosef I. Manor M. Kiro R. Qimron U. (2015). Temperate and lytic bacteriophages programmed to sensitize and kill antibiotic-resistant bacteria. *Proc. Natl. Acad. Sci. U.S.A.* 112 7267–7272. 10.1073/pnas.1500107112 26060300 PMC4466736

[B127] Yu X. Chen F. Wang R. Li Y. (2018). Whole-bacterium SELEX of DNA aptamers for rapid detection of *E.coli O*157:H7 using a QCM sensor. *J. Biotechnol.* 266 39–49. 10.1016/j.jbiotec.2017.12.011 29242148

[B128] Yunn N. O. Koh A. Han S. Lim J. H. Park S. Lee J. (2015). Agonistic aptamer to the insulin receptor leads to biased signaling and functional selectivity through allosteric modulation. *Nucleic Acids Res.* 43 7688–7701. 10.1093/nar/gkv767 26245346 PMC4652772

[B129] Zankari E. Hasman H. Cosentino S. Vestergaard M. Rasmussen S. Lund O. (2012). Identification of acquired antimicrobial resistance genes. *J. Antimicrob. Chemother.* 67 2640–2644. 10.1093/jac/dks261 22782487 PMC3468078

[B130] Zhang J. Liu J. Yang W. Cui M. Dai B. Dong Y. (2019). Comparison of gene editing efficiencies of CRISPR/Cas9 and TALEN for generation of MSTN knock-out cashmere goats. *Theriogenology* 132 1–11. 10.1016/j.theriogenology.2019.03.029 30981084

[B131] Zhang X. Feng Y. Yao Q. He F. (2017). Selection of a new *Mycobacterium tuberculosis* H37Rv aptamer and its application in the construction of a SWCNT/aptamer/Au-IDE MSPQC H37Rv sensor. *Biosens. Bioelectron.* 98 261–266. 10.1016/j.bios.2017.05.043 28689112

[B132] Zhang Y. Luo F. Zhang Y. Zhu L. Li Y. Zhao S. (2018). A sensitive assay based on specific aptamer binding for the detection of *Salmonella enterica* serovar Typhimurium in milk samples by microchip capillary electrophoresis. *J. Chromatogr. A* 1534 188–194. 10.1016/j.chroma.2017.12.054 29289340

[B133] Zhao J. Inomata R. Kato Y. Miyagishi M. (2020). Development of aptamer-based inhibitors for CRISPR/Cas system. *Nucleic Acids Res.* 10.1093/nar/gkaa865 Online ahead of print. 33123724 PMC7897479

[B134] Zhao M. Li W. Liu K. Li H. Lan X. (2019). C4-HSL aptamers for blocking qurom sensing and inhibiting biofilm formation in *Pseudomonas aeruginosa* and its structure prediction and analysis. *PLoS One* 14:e0212041. 10.1371/journal.pone.0212041 30779754 PMC6380626

[B135] Zhou J. Satheesan S. Li H. Weinberg M. S. Morris K. V. Burnett J. C. (2015). Cell-specific RNA aptamer against human CCR5 specifically targets HIV-1 susceptible cells and inhibits HIV-1 infectivity. *Chem. Biol.* 22 379–390. 10.1016/j.chembiol.2015.01.005 25754473 PMC4369413

[B136] Zhuang J. Tan J. Wu C. Zhang J. Liu T. Fan C. (2020). Extracellular vesicles engineered with valency-controlled DNA nanostructures deliver CRISPR/Cas9 system for gene therapy. *Nucleic Acids Res.* 48 8870–8882. 10.1093/nar/gkaa683 32810272 PMC7498310

